# How the Experience of Emotion is Modulated by Facial Feedback

**DOI:** 10.1007/s10919-017-0264-1

**Published:** 2017-09-27

**Authors:** Sven Söderkvist, Kajsa Ohlén, Ulf Dimberg

**Affiliations:** Department of Psychology, Uppsala University, Box 1225, 751 42 Uppsala, Sweden

**Keywords:** Facial feedback, Facial expressions, Emotion, Emotional stimuli

## Abstract

The facial feedback hypothesis states that facial actions modulate subjective experiences of emotion. Using the voluntary facial action technique, in which the participants react with instruction induced smiles and frowns when exposed to positive and negative emotional pictures and then rate the pleasantness of these stimuli, four questions were addressed in the present study. The results in Experiment 1 demonstrated a feedback effect because participants experienced the stimuli as more pleasant during smiling as compared to when frowning. However, this effect was present only *during* the critical actions of smiling and frowning, with no remaining effects after 5 min or after 1 day. In Experiment 2, feedback effects were found only when the facial action (smile/frown) was incongruent with the presented emotion (positive/negative), demonstrating attenuating but not enhancing modulation. Finally, no difference in the *intensity* of produced feedback effect was found between smiling and frowning, and no difference in feedback effect was found between positive and negative emotions. In conclusion, facial feedback appears to occur mainly during actual facial actions, and primarily attenuate ongoing emotional states.

## Introduction

The facial feedback hypothesis is based on the idea that a facial expression not only expresses an emotion, but also that expression and experience are linked in that afferent sensory feedback from the facial action influences the emotional experience. Elevating your cheeks can make you happier, just as furrowing your brow can make you angrier. The idea was introduced by Darwin (1872) when he noted that the experience of an emotion seemed to be intensified when the emotion was freely expressed, but softened when repressed. James (1884) also presented ideas along the same lines, but it took almost a century before the specific facial feedback hypothesis was formulated by Tomkins (1962), Gellhorn (1964), Izard (1971), Ekman (1973) and Buck (1980). Recently, Niedenthal (2007) and (Niedenthal et al. 2009, 2010) provided the theoretical framework of embodied emotion to explain, among other things, afferent feedback from facial actions. Empirical research has found that facial feedback has the ability to both modulate present emotions and to initiate emotions (for reviews see Adelmann and Zajonc 1989; Buck 1980; McIntosh 1996). This has been firmly established for happiness and anger and the corresponding facial expressions (e.g., Dimberg and Söderkvist 2011; Laird 1974; Rutledge and Hupka 1985). Also, feedback mechanisms seem involved in the expression of fear and sadness (e.g., Flack et al. 1999), as well as for surprise and disgust (e.g., Lewis 2012).

Recent facial feedback research has sought to improve knowledge of basic mechanisms of facial feedback, and attempted to demonstrate clinical benefits from manipulating the facial muscles. One insight to the basic mechanisms has been provided by Hennenlotter et al. (2009), who demonstrated that facial feedback modulates neural activity in the amygdala. Using fMRI they found that amygdala activation was attenuated during imitation of angry facial expressions in participants with Botox-induced paralysis of their corrugator muscles, the muscles used when frowning. Kim et al. (2014) further demonstrated that facial feedback modulates activity in the amygdala in an fMRI study where participants were passively exposed to happy and angry facial expressions. Amygdala activity was attenuated when corrugator muscles were paralyzed by Botox, as compared to tests before injections and also to tests after the effects had worn off.

Furthermore, Mori and Mori (2010, 2013) have demonstrated significant effects from a passive form of facial feedback, where facial expressions were formed, without any muscle action, on the face of participants with the help of elastic adhesive bandages. Interestingly, since no muscles were activated, any feedback must have originated from sensations in the skin or from proprioceptive signals in the cutaneous muscles. Moreover, Dimberg and Söderkvist (2011) demonstrated that facial feedback was more effective at modulating emotions, as compared to initiating emotions, and that feedback was equally effective at modulating both positive and negative emotions. That study also found that effects of facial feedback actions could be detected several minutes later, a result that calls for further research.

Clinical benefits from manipulating facial muscles have been demonstrated in several studies, where Botox injections were used to paralyze the corrugator muscles of clinically depressed patients. The results showed significant mood improvements in patients, and these effects were attributed to reduced facial feedback from the corrugator muscles (Finzi and Rosenthal 2014; Magid et al. 2014; Wollmer et al. 2012, 2014).

Yet, it remains unclear *how* facial feedback actually works. The most commonly held theory states that when certain facial muscles are activated, afferent feedback from muscular proprioceptive patterns activates corresponding affect programs (Adelmann and Zajonc 1989; Tomkins 1962). However, sensory feedback from the facial skin has also been proposed as a possible contributor to facial feedback (Tomkins 1980). Furthermore, the vascular theory of emotional efference provides another explanation suggesting that facial actions can regulate brain blood temperature by altering nasal air flow, which in turn could produce changes in emotional state (Zajonc et al. 1989). However, it is inherently difficult to identify the neurological basis of the above theories and it also follows that it is difficult to specifically test the theories. Alternatively, one way forward could be to further explore different effects of facial feedback and in this way improve the description of how facial feedback effects occur and thereby extend the boundary conditions of the hypothesis.

Therefore, in two experiments the present study addressed a series of questions that together could provide a more detailed description of how facial feedback effects occur. The two experiments used *the voluntary facial action technique,* which was developed in Dimberg and Söderkvist (2011), based on the method used in Dimberg et al. (2002). In this paradigm participants are told a cover story that the purpose of the experiment is to measure the reaction times of different facial muscles. The cover story has proved effective in hiding the true purpose of the manipulation, which in fact is to get the participants to perform particular facial actions associated with specific emotions. In facial feedback experiments it is common to use a cover story in order to lead the participants’ attention away from facial expressions and emotions to ensure that they are unconsciously affected by the feedback, which also serves to avoid demand characteristics. In the present paradigm participants are then required to react with their zygomatic muscles, which are the muscles used when smiling, and/or corrugator muscles, which are used when frowning, to different kinds of pictures, which encourage facial actions that resemble naturally occurring facial expressions. When testing the modulating ability of facial feedback, emotion evoking stimuli, such as pictures of happy and angry faces, have been successfully used (Adelmann and Zajonc 1989; Dimberg and Söderkvist 2011; McIntosh 1996). While reacting to the pictures, the participants also rate how pleasant/unpleasant they find them, and after completion each participant has thus smiled one time and frowned one time at each picture. If they rate the pictures as more pleasant and less unpleasant when they smile, as compared to when they frown, a general facial feedback effect has been demonstrated.

In Experiment 1, a question first raised by Dimberg and Söderkvist (2011) was further investigated, and focused on whether facial feedback actions only affect us when they are performed, or if the effect also remains to affect us later.

In Experiment 2, three questions were addressed. First, does facial feedback primarily enhance or attenuate present emotions? We know that facial feedback can both enhance and attenuate emotions, but it has not previously been specifically investigated if feedback is more effective in modulation where the facial action is congruent with the present state to enhance it, or if it is more effective in incongruent modulation to attenuate the emotion. The second question was if smiling or frowning generally produces a stronger feedback effect than the other, or if they produce equally strong feedback effects. It is well established that these two major facial actions both produce facial feedback, but it remains unclear if they are equally effective. Third, another question that was also first addressed in Dimberg and Söderkvist (2011) was if positive or negative emotions are easier to modulate with facial feedback or if both are modulated equally effective by the feedback.

## Experiment 1

The aim of the first experiment was to further investigate if the effect of facial feedback actions could remain and also affect us later. In Dimberg and Söderkvist (2011), a significant effect of facial feedback was found about 4 min after the facial action. The design in that experiment, based on the voluntary facial action technique, let participants in a first phase react with smiles/frowns to pictures with positive or negative valence, without rating the pictures. In a second phase they viewed the pictures once again, and this time they did not voluntary move their facial muscles but just rated how pleasant/unpleasant they found the pictures. A facial feedback effect was demonstrated, because the rating of a picture in the second phase was affected by which facial action the participant had performed to that picture in the first phase. That is, participants found pictures to be more pleasant if they had smiled to them 4 min earlier, than if they had frowned.

However, the experiment by Dimberg and Söderkvist (2011) had some limitations. Specifically, no effects were measured during the facial actions in the experiment, and no effects later than 4 min were evaluated. It is therefore unclear if the intensity of the effect had changed during the 4 min, and it is also unclear for how long the effect remained. In order to investigate this question more extensively, the present Experiment 1 was designed to measure the facial feedback effects both *during* the facial actions, 5 min after the actions and approximately 1 day after the actions. The procedure and cover story in the present experiment was essentially the same as the above presented voluntary facial action technique, but some changes were introduced in order to facilitate the measurement of effects at the three different points in time.

If feedback effects would be detected after the initial action, they could be explained in terms of embodied emotion (e.g., Niedenthal 2007) and/or evaluative conditioning (e.g., Hofmann et al. 2010). Considering the empirical literature on facial feedback, a feedback effect during the facial actions was expected. Based on the results in Dimberg and Söderkvist (2011), a feedback effect after 5 min could be expected as well. However, we had no specific expectations for the outcome after 1 day since this has not been investigated before.

## Method

### Participants

Thirty-two persons (mean age = 25.40, *SD* = 5.20), balanced by gender, participated in the experiment and most of them were students at Uppsala University. No one that studied psychology or previously had participated in similar studies was included. Five persons were excluded from the study due to not following the instructions correctly, and they were replaced by other participants. As compensation, participants were given three movie vouchers at a total value of 300 SEK (approximately 30 USD).

### Experimental Design

Because the aim of the experiment was to examine facial feedback effects over time, the two main independent variables were muscle (smile vs. frown) and time (during action vs. after 5 min vs. after 1 day). A secondary independent variable was stimulus (happy vs. angry). Thus, the experiment had a within subject 2 × 3 × 2 factorial design. The dependent variable was the ratings of how pleasant/unpleasant participants experienced stimuli.

### Apparatus

Much of the apparatus served only a deceptive purpose in order to strengthen the cover story. The psychophysiological laboratory, for example, played an important role for the credibility of the cover story. The participants were individually tested in a small room inside the laboratory, where they were seated in a comfortable chair with a 17 inch computer monitor placed 70 cm in front of them. A PC placed outside the room was used to display the pictures. In accordance with the cover story, electrodes to ostensibly measure EMG were attached over the *corrugator supercilii* (frowning) and the *zygomatic major* (smiling) muscle regions.

The stimulus material consisted of six pictures of happy faces and six pictures of angry faces, selected from Ekman and Friesen’s *Pictures of facial affect* (1976). Even if some studies (e.g., Wangelin et al. 2012) have demonstrated that pictures of facial expressions do not typically induce strong emotional engagement, other studies performed with similar exposure techniques and experimental situations (e.g., Dimberg 1982, 1990; Dimberg et al. 1998, 2000, 2002) have demonstrated that pictures of facial stimuli can reliably induce positive and negative emotions of equal intensity. One further reason for using these stimuli was to maintain consistency with Dimberg and Söderkvist (2011), where the same material were used. The pictures in the present study were presented in the form of PowerPoint slideshows, where each picture was shown for 8 s and then followed by a black screen for 20, 25 or 30 s before the next picture. Participants rated picture experience using the two categories pleasantness and unpleasantness on a scale from 0 to 100 (*not at all* to *very much*). The reason for using two opposing scales was that we could not rule out the possibility that they would be differently sensitive to the different muscle and stimulus conditions in the experiment. For each picture, a sheet containing both scales was used.

As part of a distraction task, performed between experimental phases in order to prevent carry-over effects, participants pressed a button on a handheld device to seemingly register their reaction time in response to displayed pictures of circles and triangles, three of each.

### Cover Story

The cover story was essentially the same as in Dimberg and Söderkvist (2011), with a couple of small adjustments for the new procedure. Information on signup posters and in scheduling emails had briefed the participants that the experiment would measure reaction times in different muscles and to different kinds of stimuli. At arrival, they were given a more detailed introduction to the experiment, including a presentation of the psychophysiological apparatus. The experiment was presented as the latest in a run of experiments that aimed to clarify how fast human beings could react. The claimed purposes of this specific experiment were to (first) compare the reaction times in facial muscles with the more traditional reaction time measurement of pressing a button with your thumb, and (second) to see how much the reaction time varied over different days. The stimulus material was presented as pictures of human faces and of geometrical figures and the reason for using them was to compare with previous experiments that had used other kinds of stimuli, like audio signals. Participants were told that the reason for rating the pleasantness and unpleasantness of the stimuli was to control for the possibility that the reaction time is affected by how we like the picture. The purpose of this explanation was to mislead participants that might suspect a hidden agenda into believing that the agenda was in fact to see if different kinds of pictures affected the reaction time.

### Procedure

**Fig. 1:**
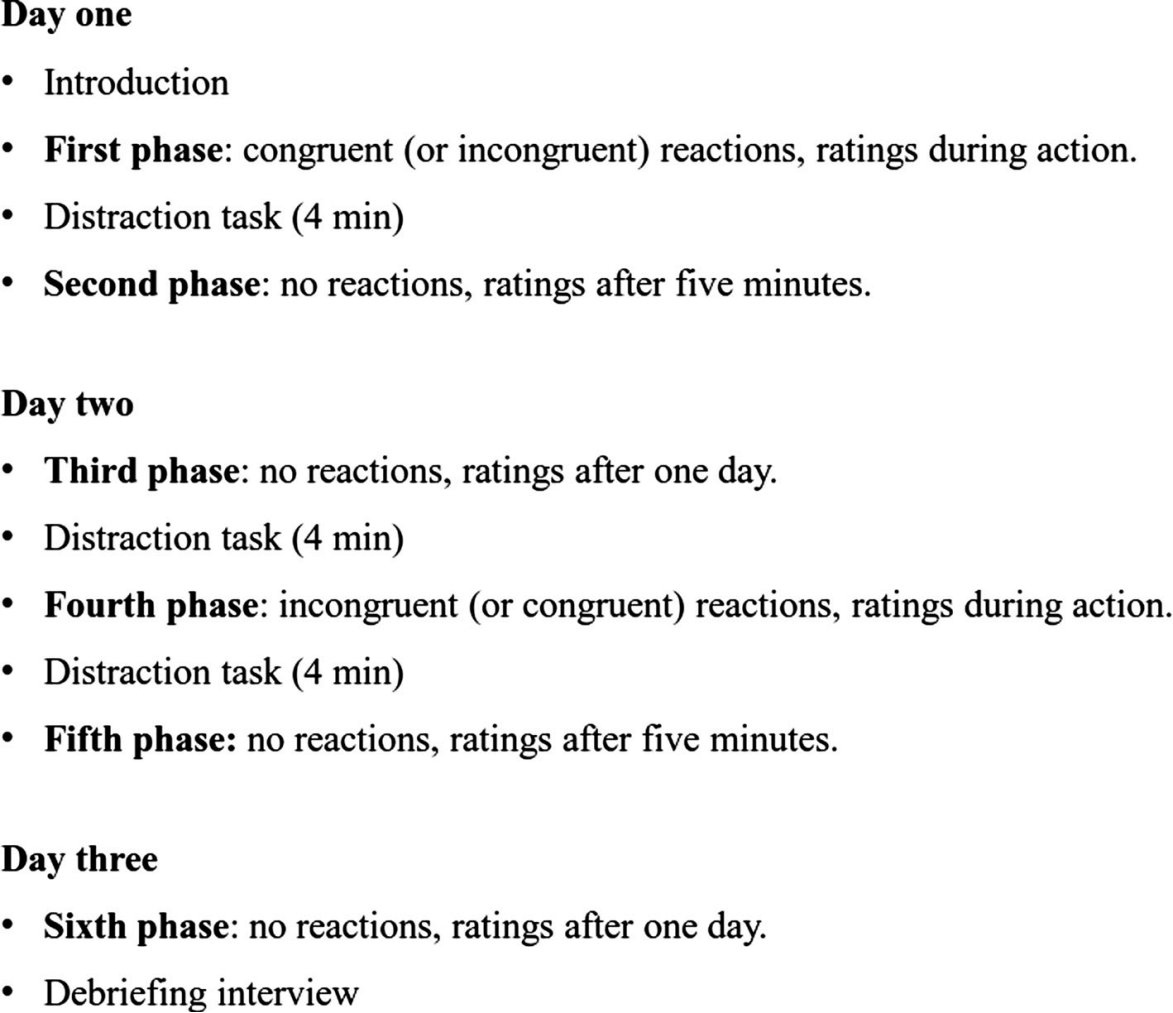
The procedure over three separate testing days for each participant

Participants attended the laboratory on three separate days within the same workweek. They performed two nearly identical cycles of testing (congruent/incongruent reactions) and in each cycle they rated the stimuli at three different points in time (during action, after 5 min, after 1 day). Half of the participants began with the congruent reactions, and the other half began with the incongruent reactions. In the congruent cycle they were instructed to react by elevating their cheeks as fast as possible if the displayed picture was of a happy person, and to contract their eyebrows as fast as possible if the picture was of an angry person. In the incongruent cycle they received the reversed instructions and thus elevated their cheeks to angry persons and contracted their eyebrows to happy persons. Consequently, and as can be seen in Fig. 1, the procedure consisted of six different phases: 1—congruent (or incongruent) reactions, ratings during action, 2—no reactions, ratings after 5 min, 3—no reactions, ratings after 1 day, and then a new cycle started with 4—incongruent (or congruent) reactions, ratings during action, 5—no reactions, ratings after 5 min, and 6—no reactions, ratings after 1 day. Additionally, phases performed during the same day had distraction tasks between them to prevent carry-over effects.

#### Day One

After the initial presentation of the experiment, the first phase began (congruent or incongruent reactions, ratings during action). The participants were asked to sit down in the smaller room where the electrodes were attached on their face. Half of them were randomized to receive the congruent instructions for how to react, while the other half received incongruent instructions. All were told that in order for the apparatus to register their reaction time properly they needed to make a distinct muscle contraction and keep it contracted for a couple of seconds. Finally, they were introduced to the rating scales and instructed to rate for each picture, during the display, how pleasant and unpleasant they experienced it. They were then left alone and exposed to all the six happy and six angry pictures.

After this first phase, a 4 min distraction task followed where they first received instructions for how and when to use the handheld device. Half of the participants were instructed to press the button if the displayed picture was of a circle, and to do nothing if it was of a triangle. The other half received the opposite instructions, to press the button if the picture was of a triangle and to do nothing if it was of a circle. During this task they did not rate the displayed pictures. All subsequent distraction tasks had similar structure.

After the distraction task, the second phase (no reactions, ratings after 5 min) followed. The participants received instructions that they would see the pictures of human faces once again, but this time their reaction time would not be measured. Instead they were instructed to keep their facial muscles still and relaxed. New rating sheets were handed to them together with a repetition of the rating instructions. The stated reason for this extra rating session was to ensure stable and valid measurements. Participants were then exposed to the pictures in a different order as compared to the first phase.

#### Day Two

When the participants returned for day two, the third phase began (no reactions, ratings after 1 day). They were again asked to sit down in the smaller room and the electrodes were attached. They were then informed that the day would begin with a rating session similar to the one that ended day one, where they did not react with their facial muscles. Rating sheets were handed out and they were exposed to the pictures in a different order compared to the first and second phase.

After a distraction task, the fourth phase began (incongruent or congruent reactions, ratings during action) and it was identical to the first phase, but with the critical muscle instructions reversed. After another distraction task, the fifth phase followed (no reactions, ratings after 5 min), which was identical to the second phase.

#### Day Three

Just as in previous days, all participants were asked to sit down in the smaller room while electrodes were attached. This sixth and final phase (no reactions, ratings after 1 day) was identical to the third phase, and picture ratings were performed with muscles relaxed.

After the rating session, participants were informed that they had completed the experiment. They were then interviewed in order to determine if they had followed the instructions correctly, and if they at any point had realized the true purpose of the experiment, which none of them had. Finally, they were informed about the true purpose and were asked to keep it secret in conversation with others.

### Transformations and Analysis

As a result of the experimental procedure, each participant had rated how pleasant and unpleasant they found the six happy and six angry pictures during smiling respectively frowning within each of the three different conditions of time (during action, after 5 min, after 1 day). Similar to the procedure in earlier studies (Dimberg and Söderkvist 2011) we compared the pleasantness and unpleasantness ratings in a preliminary analysis to determine if there were any major differences between them. When we correlated the two ratings within each participant the median correlation coefficient for all participants was −0.85 (*SIQR* = 0.11), indicating that the two measures seemed to reflect the same emotional quality. Furthermore, the results provided by the pleasantness and the unpleasantness ratings pointed in the same direction and the interpretations of them were quite similar. Consequently, to provide a more distinct presentation of the results we therefore merged the two ratings into a single bipolar rating score by subtracting the unpleasantness ratings from the pleasantness ratings. The resulting bipolar pleasantness rating scale ranged from −100 to 100, with a positive value thus indicating pleasantness, and a negative unpleasantness.

Because the purpose of the study was to measure and compare the magnitude of facial feedback effect at different points in time a new dependent variable specifically representing this was created. The *facial feedback effect* (*FFE*) *score* was calculated for each rated picture by subtracting the frown rating from the corresponding smile rating within each condition of time. A positive value in the resulting score thus indicated a facial feedback effect, since the picture had been rated as more pleasant during or after the smile, as compared to during or after the frown. Also, for each participant, the ratings of the six happy and the six angry pictures within each condition of time were collapsed into one mean for the happy and one mean for the angry pictures. Consequently, each participant had six mean FFE scores, with one score during positive emotions (to happy pictures) and one score during negative emotions (to angry pictures) for each of the three conditions in time. To analyze the data a 3 × 2 within subject ANOVA was performed, with the factors time (during action vs. after 5 min vs. after 1 day) and emotion (positive vs. negative). The significance level was set to 0.05 for all tests.

## Results

A first analysis revealed that there was no main effect of emotion and no interaction between emotion and the time factor, both *F*:s < 1. Therefore, to simplify the analysis and the presentation of results, the data for positive and negative emotions were collapsed so that in the final analysis only the mean FFE scores as a function of time are reported.

**Fig. 2:**
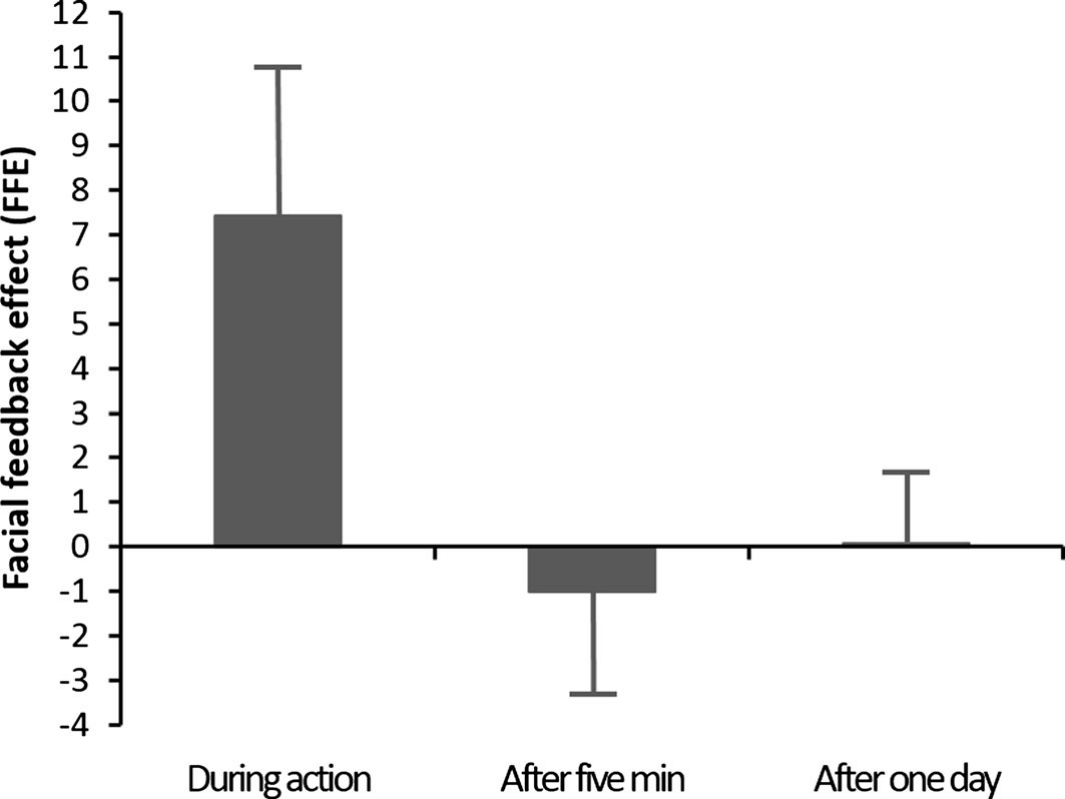
Mean facial feedback effect (FFE) score during action, after 5 min, and after 1 day, with error bars representing one standard error

There was a significant main effect of time, *F*(2,62) = 4.57, *MS*_e_ = 147.12, *p* < 0.05, *η*_*p*_^*2*^ = 0.13, and as can be seen in Fig. 2 this was due to a much higher mean FFE score during action, as compared to the scores after 5 min and after 1 day. Subsequent *t*-tests further confirmed that the mean FFE score during action [*M* = 7.42, *SD* = 19.28, 95% CI (0.47, 14.37)] was significantly higher than the score after 5 min [*M* = −1.01, *SD* = 12.84, 95% CI (−5.64, 3.62)], *t*(31) = 2.78, *p* < 0.05, *d* = 0.69, and after 1 day [*M* = 0.08, *SD* = 9.40, 95% CI (−3.31, 3.47)], *t*(31) = 2.42, *p* < 0.05, *d* = 0.61. Additional one-sample *t*-tests also demonstrated that only the mean FFE score during action differed significantly from zero, *t*(31) = 3.46, *p* < 0.05, *d* = 0.61, while the mean FFE scores after 5 min and after 1 day did not differ from zero, both *t*:s < 1. That is, facial feedback effects were demonstrated only during action, but no remaining effects during the two later phases.

## Discussion

The aim of the experiment was to investigate if the effect of facial feedback actions remained to affect us at a later time. As expected, and consistent with earlier findings (Adelmann and Zajonc 1989; Dimberg and Söderkvist 2011; McIntosh 1996), there was a significant facial feedback effect, with a medium to large effect size, during the facial action. However, there were no effects after 5 min or 1 day. Thus, the present experiment did not replicate the result of the previous experiment by Dimberg and Söderkvist (2011), where an effect of facial feedback was detected about 4 min after the facial action. The two experiments had slightly different designs though, where the present experiment measured the effect at three different points in time and also included specific distraction tasks. It is possible that these differences could account for the diverging results, and the most likely explanation would then be that any remaining effects were abolished by the distraction tasks. If that is the case, it would suggest an initially strong and influential effect of facial feedback, but an effect that is also short-lived and easily dissipated once the facial action is over. The present results interpreted in terms of embodied emotion (e.g., Niedenthal 2007) would suggest that the reactivation of the facial action performed at the first exposure was not strong enough to affect the experience at the later exposures. Similarly, the results interpreted in terms of evaluative conditioning (e.g., Hofmann et al. 2010) would suggest that the affective signature tied to the stimulus by the facial action was not strong enough to affect the evaluation of the stimulus at later exposures.

As compared to the original voluntary facial action technique, the procedure in the present experiment spanned over several days, which presented a challenge for the cover story. Whereas participants in previous experiments only had to believe the cover story for an hour, participants in the present experiment had to believe it for half a week, and this of course increased the risk that they would realize the true purpose. However, not a single participant realized the true purpose, which further confirms that the cover story used in the voluntary facial action technique is robust and can be used in different experimental approaches and designs.

In conclusion, the results in the present Experiment 1 suggest that the effect of facial feedback primarily occurs at the same time as the facial action is performed.

## Experiment 2

In Experiment 1, there was a significant effect of facial feedback during the facial actions. However, similar to many previous studies (Adelmann and Zajonc 1989; Dimberg and Söderkvist 2011; McIntosh 1996), this feedback effect was calculated as the difference in ratings between smiling actions and frowning actions. That is, without any ratings during a neutral action, to which the smiling and frowning ratings could be separately compared, it is not possible to conclude to what degree this feedback effect was the result of enhancing or attenuating modulation, and if smiling or frowning produced stronger effect than the other. Therefore, Experiment 2 introduced a neutral action (see below) with the aim to address these questions and provide a more detailed description of how facial feedback effects occur. The experiment addressed three different questions and they will first of all be presented in more detail.

*The first question* was if facial feedback primarily enhances or attenuates a present emotion. For example, with a positive emotion present, such as during exposure to pictures of happy faces, smiling could further enhance positivity, while frowning could attenuate it. And in contrast, with a negative emotion present, such as during exposure to pictures of angry faces, smiling could attenuate negativity, while frowning could further enhance it. That is, enhancing modulation consists of both smiling during positive emotions and frowning during negative emotions, while attenuating modulation consists both of frowning during positive emotions and smiling during negative emotions. In order to determine if facial feedback primarily enhances or attenuates emotions the magnitude of facial feedback effect in the enhancing respectively the attenuating modulation need to be measured and compared, which means that we need to determine the *specific* feedback effects of smiling and frowning. As mentioned above, with the help of a neutral muscle condition these effects can be calculated by separately comparing the ratings when smiling and the ratings when frowning to the ratings in a neutral condition. For example, there would be a specific feedback effect of *smiling* if the ratings when smiling were *higher* than the ratings in a neutral condition, since feedback from a smile is expected to *increase* the ratings of pleasantness. On the other hand, there would be a specific feedback effect of *frowning* if the ratings when frowning were *lower* than the ratings in a neutral condition, since feedback from a frown is expected to *decrease* the ratings of pleasantness. Consequently, it would also be possible to conclude if the feedback effect of smiling is stronger with a positive emotion present (enhancing) or with a negative emotion present (attenuating), and if the feedback effect of frowning is stronger with a negative emotion present (enhancing) or with a positive emotion present (attenuating).

In the literature on facial feedback, both enhancing and attenuating modulation have been demonstrated, but there are no studies, as far as we know, that have compared the two in a manner that could investigate if feedback is more effective to enhance or attenuate emotions (Adelmann and Zajonc 1989; McIntosh 1996). Even though many of the first studies on facial feedback included neutral muscle conditions (e.g., Laird 1974; Rutledge and Hupka 1985), their focus was primarily on testing the main hypothesis of facial feedback, and studies were typically not designed or analyzed in a way that could answer the present questions at hand.

In a classical study by Strack et al. (1988), a neutral muscle condition was included in a design where participants were instructed to rate the funniness of cartoons with the help of a pen either held between the teeth, between the lips or in the non-dominant hand. Holding the pen between the teeth facilitated smiling since the participants needed to activate the *zygomaticus major* muscle in order to keep the lips from the pen. Holding the pen between the lips, on the other hand, inhibited smiling and also activated the *orbicularis oris* muscle, involved in the expression of anger (Ekman and Friesen 1975; Hjortsjö 1970). Holding the pen in the non-dominant hand was in this case the neutral muscle condition. The results demonstrated significant differences, where the funniness ratings were highest for participants who had been facilitated to smile, lowest for participants who had been inhibited to smile, and with the neutral condition perfectly placed in the middle. However, a recent attempt to directly replicate this experiment found no effects on the funniness ratings at all between the teeth and the lip positions of the pen (Wagenmakers et al. 2016). The absence of effects was unexpected since numerous studies over the years have used variations of the original method to successfully manipulate emotions, even if no direct replication had been done previously (Strack 2016).

Even though the present-day effectiveness of the original method must be questioned in light of the failed replication, the original study by Strack et al. (1988) still remains of interest for the present study since it demonstrated both enhancing and attenuating feedback effects. That is, with a positive emotion present, a smile enhanced the rated funniness while a mouth that expressed anger attenuated the rated funniness. In other words, the original results demonstrated that facial feedback was equally effective at enhancing and attenuating a positive emotion in this setting. However, it is necessary to note that holding a pen between the lips, and thereby activating the *orbicularis oris*, does not constitute a complete and valid expression of anger (Ekman and Friesen 1975; Hjortsjö 1970). An essential part of expressing anger is to frown and this action was not included in the study by Strack et al. (1988), and by extension not in the replication effort (Wagenmakers et al. 2016). Therefore, it is unclear if it is effective enough to compare these two muscle groups to each other, since it is possible that the rated funniness had been lower if the participants had also been instructed to frown to the cartoons. In summary, the original study by Strack et al. (1988) demonstrated that facial feedback had the ability to both enhance and attenuate present positive emotions, even though their relative effectiveness remained unknown. Note however that this result could not be replicated by Wagenmakers et al. (2016).

Soussignan (2002) adopted almost the same method as Strack et al. (1988), but with two separate intensity levels of smiling, one low and one high. The study had both positive and negative emotional stimuli, and the neutral muscle condition was to hold the pen in the mouth in a way that relaxed most muscles. The results showed that the high intensive smile group had significantly higher ratings than the other groups, but only to positive emotional stimuli. There were no feedback effects at all to negative stimuli. Furthermore, there was no difference in ratings between the group that had the pen between the lips and the neutral control group. That is, with a positive emotion present, a smile enhanced the ratings, but a mouth that expressed anger did not attenuate them, a result suggesting that facial feedback primarily enhances present positive emotions. However, since the study by Soussignan (2002) used a similar method to Strack et al. (1988), the same limitations apply to the angry mouth condition, which means that an attenuating effect remained a possibility even though no effects were found.

Thus, the present study addressed the question if facial feedback primarily enhances or attenuates emotions by introducing two neutral muscle control conditions to the voluntary facial action technique. In line with the cover story of measuring reaction times, the neutral conditions were to press a button on a handheld device and/or to do nothing. Consequently, the participants would ostensibly test their reaction times in two different phases: one facial phase where they would react with either a smile or a frown to pictures of happy and angry faces, and one neutral phase where they would react by either pressing a button or doing nothing to the same set of pictures. By comparing the effects of the smile and the frown conditions with the neutral conditions, it would then be possible to evaluate (1) if facial feedback primarily enhances emotions, a situation where smiles enhance positive emotions and frowns enhance negative emotions, or (2) if feedback primarily attenuates emotions, a situation where smiles attenuate negative emotions and frowns attenuate positive emotions, or (3) if feedback both enhances and attenuates emotions.

*The second question,* if smiling or frowning produces a stronger feedback effect than the other, is closely related to the first question. The happy smile and the angry frown are powerful emotional expressions and it is well established that both induce facial feedback. However, it remains unknown if smiling and frowning are equally effective when it comes to modulating our present emotions. In Experiment 1, as well as in Dimberg and Söderkvist (2011), a general feedback effect was calculated by the difference in ratings between smiling and frowning conditions. With such procedure however it is not possible to tell if the feedback comes from smiling, from frowning, or from both. Thus, as in the above first question, a neutral muscle condition is necessary in order to discern the specific feedback effects of smiling and frowning. Consequently, the present study addressed this question with the help of the two neutral muscle conditions described above.

*The third question,* if positive or negative emotions are easier to modulate with feedback, was addressed by Dimberg and Söderkvist (2011) who found no difference between positive and negative emotions in regards to feedback effects. However, in the study by Soussignan (2002) mentioned above, significant feedback effects were only found during positive stimuli, with no effects during negative stimuli. Moreover, and in contrast, a study by Davis et al. (2009) found feedback effects during negative stimuli, but not during positive, when they compared inhibited and non-inhibited facial reactions to emotional stimuli. The aim of the present study was to further investigate this question. Note that this third question is intimately related to the first and second questions in the sense that some specific outcomes of those two questions could provide the answer to this question. For example, if facial feedback primarily enhances present emotions, at the same time as feedback effects from smiling are stronger, then it follows that facial feedback modulates positive emotions more effectively. Considering that previous studies have demonstrated inconsistent results, the present study had no specific expectations regarding the outcome.

## Method

### Participants

Sixty-four persons (mean age = 23.75, *SD* = 3.98), balanced by gender, participated in the experiment and most of them were students at Uppsala University. No one that studied psychology or previously had participated in similar studies was included. Five persons were excluded from the study due to not following the instructions correctly, and five persons were excluded due to realizing the true purpose of the study, and they were all replaced by other participants. As compensation, participants were given a movie voucher at a value of 100 SEK (approximately 10 USD).

### Experimental Design

The two independent variables of the experiment were muscle (smile vs. frown vs. button vs. nothing) and stimulus (happy vs. angry), and it thus had a within subject 4 × 2 factorial design. The dependent variable was the ratings of how pleasant/unpleasant the participants experienced the stimuli.

### Apparatus

Similar to the procedure in Experiment 1, the participants were individually tested in a small room inside the psychophysiological laboratory, where they were seated in front of a computer monitor. In the facial phase, where they reacted with either a smile or a frown, electrodes to ostensibly measure EMG were attached over the *zygomatic major* (smiling) and the *corrugator supercilii* (frowning) muscle regions. In the neutral phase, where they reacted by either pressing a button or doing nothing, a handheld device was used to seemingly register their reaction times.

The stimulus material consisted of the same six pictures of happy faces and six pictures of angry faces (Ekman and Friesen 1976) that were successfully used in Experiment 1, and another reason for using these stimuli was once again to maintain consistency with the experiments in Dimberg and Söderkvist (2011). The pictures were presented in the form of PowerPoint slideshows, where each picture was displayed for 8 s and then followed by a black screen for 20, 25 or 30 s. Participants rated on two different scales of 0–100 (*not at all* to *very much*) how pleasant and unpleasant they experienced the pictures. For each picture, a sheet containing both scales was used.

### Procedure

As with the cover story in Experiment 1, participants were told that the aim of the experiment was to measure the reaction times in different muscles. Half of the participants were randomized to begin with the facial phase, where they had electrodes attached on their facial muscles and were instructed to react with either their smile or their frown muscles. After a short break, they continued with the neutral phase where they were introduced to the handheld device and were instructed to react either by pressing a button on the device or by doing nothing. The other half of participants began with the neutral phase, and then continued with the facial phase after a short break. Two different neutral conditions were needed in order for the neutral phase to be as similar as possible to the facial phase, and to seem a plausible test of reaction times.

Within each phase, the participants performed two different rating tasks. In each of these tasks they were exposed to all the six happy and six angry pictures, in different orders, and were instructed to rate how pleasant and unpleasant they experienced them. In the facial phase, one task was to react by elevating the cheeks as fast as possible if the displayed picture was of a happy person, and to contract the eyebrows as fast as possible if the picture was of an angry person. The other task was accompanied by the reversed instruction, that is, to elevate the cheeks if the picture was of an angry person and to contract the eyebrows if the picture was of a happy person. The order of tasks was balanced amongst the participants. In the neutral phase, one task was to react by pressing a button on the handheld device as fast as possible if the displayed picture was of a happy person, and to do nothing if the picture was of an angry person. The other task was with the reversed instructions, that is, to press a button on the handheld device if the picture was of an angry person and to do nothing if the picture was of a happy person. After the experiment, participants were interviewed in order to determine if they had followed the instructions correctly and if they at any time had realized the true purpose of the experiment.

### Transformations and Analysis

As a result of the procedure, each participant had rated how pleasant and unpleasant they found the six happy and six angry pictures during the four different muscle conditions: smiling, frowning, pressing a button, and doing nothing. Before further analysis, similar to the procedure in Experiment 1 and earlier studies (Dimberg and Söderkvist 2011), we compared the pleasantness and unpleasantness ratings to see if there were any major differences between them. When we correlated the two ratings within each participant the median correlation coefficient for all participants was −0.80 (*SIQR* = 0.13), and the results provided by the pleasantness and the unpleasantness ratings pointed in the same direction. As in Experiment 1, we therefore merged the two ratings to a single rating score by subtracting the unpleasantness ratings from the pleasantness ratings, resulting in the same bipolar scale measuring from −100 to 100 used in Experiment 1. Moreover, the ratings of the two neutral muscle conditions were compared and it was found that there was no significant difference between them, *t*(63) = 0.01, *ns*, and also that they had a strong positive correlation, *r* = 0.90, *p* < 0.05. Thus, in order to simplify the analysis, the two ratings were merged into a single neutral rating.

Because the purpose of the study was to measure and compare the magnitude of the facial feedback effects of smiling and frowning in enhancing and attenuating modulation respectively a new dependent variable specifically representing each muscle action was created. The *specific facial feedback effect* (SFFE) scores were calculated by comparing the ratings when smiling and the ratings when frowning to the ratings in the neutral condition. That is, the SFFE score for *smiling* was calculated by subtracting the ratings in the *neutral condition* from the ratings when *smiling*. This procedure created a score that had a positive value if the ratings when smiling were *higher* than the ratings in the neutral condition. The SFFE score for *frowning*, on the other hand, was calculated by subtracting the ratings when *frowning* from the ratings in the *neutral condition*, which created a score that had a positive value if the ratings when frowning were *lower* than the ratings in the neutral condition. To summarize, these calculations transformed the ratings into a score that expresses *absolute values of feedback effect*. That is, with this score a positive sign indicates a feedback effect for both smiling and frowning independent of if the facial action originally increased or decreased the rated pleasantness. As a first step in creating these new dependent variables, the SFFE scores for smiling and frowning were calculated for each rated picture. Secondly, for each participant the scores of the six happy and the six angry pictures were collapsed into one mean for the happy and one mean for the angry pictures, for smiling and frowning respectively. This resulted in four mean SFFE scores for each participant, that is, one score for smiling and one score for frowning during positive emotions (to happy faces), and one for smiling and one for frowning during negative emotions (to angry faces).

The three questions in the present study were first, if facial feedback primarily enhances or attenuates emotions, second, if smiling or frowning produces a stronger feedback effect than the other, and finally if positive or negative emotions are easier to modulate with feedback. In the analysis, these three questions were evaluated using three different factors, one for modulation (enhancing vs. attenuating), one for muscle (smiling vs. frowning), and one for emotion (positive vs. negative). Note however that due to inherent properties of these factors there exists an inevitable interdependency between them. For example, smiling as an enhancing modulation is only possible during a positive emotion and never during a negative emotion, while frowning as an enhancing modulation is only possible during a negative emotion. Similarly, smiling during a positive emotion is per definition an enhancing modulation and cannot be attenuating, while frowning during a positive emotion is always an attenuating modulation. That is, the muscle factor is intertwined and linked together with the modulation and emotion factors in these cases, which illustrates the fact that the conditions within each of the three factors by nature are *confounded* with each other. Thus, due to this interdependency between the factors it was not possible to simultaneously evaluate the effects in a three-factorial analysis of variance. Instead the three questions were addressed in three separate one-factorial analyses. That is, in each analysis, the four SFFE scores for each participant were collapsed in a different way into two mean scores. First, the mean score for *enhancing modulation* consisted of the SFFE scores for when participants smiled during positive emotions and frowned during negative emotions, while the mean score for *attenuating modulation* consisted of the SFFE scores for when participants smiled during negative emotions and frowned during positive emotions. Second, the mean score for *smiling* consisted of the SFFE scores for when they smiled during positive and during negative emotions, while the mean score for *frowning* consisted of the SFFE scores for when they frowned during positive and during negative emotions. Finally, the mean score for *positive emotions* consisted of the SFFE scores for when they smiled and frowned during positive emotions, while the mean score for *negative emotions* consisted of the SFFE scores for when they smiled and frowned during negative emotions.

## Results

**Fig. 3:**
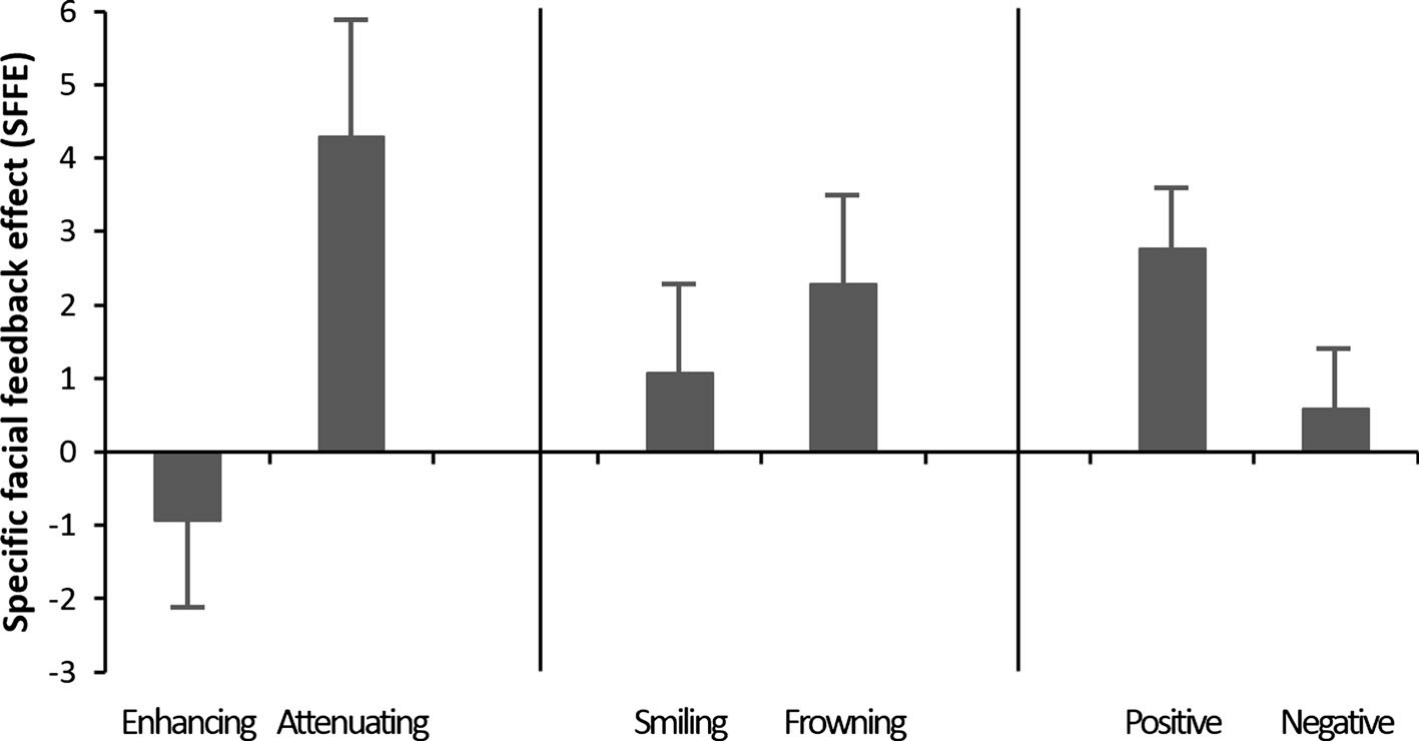
Mean specific facial feedback effect (SFFE) scores for (left) enhancing and attenuating modulation, (middle) smiling and frowning, and (right) during positive and negative emotions, with error bars representing one standard error

As explained in the method section above, the three main questions were evaluated in three separate one-factorial ANOVAs, with the data reconstructed for each analysis. The mean SFFE scores for the two conditions of each of the three factors are presented in Fig. 3.

### Enhancing Versus Attenuating

As detailed above, in order to compare the feedback effects between enhancing and attenuating modulation, the four mean SFFE scores of each participant were collapsed into two mean scores, one for enhancing and one for attenuating modulation. To analyze the resulting data, a one-way within subject ANOVA with the factor modulation (enhancing vs. attenuating) was performed. The result demonstrated a significant effect of modulation, *F*(1,63) = 4.15, *MS*_e_ = 210.58, *p* < 0.05, *η*_*p*_^*2*^ = 0.06, and as seen in the left panel of Fig. 3 this effect was due to higher SFFE scores in attenuating [*M* = 4.29, *SD* = 12.45, 95% CI (1.18, 7.40)] as compared to enhancing modulation [*M* = −0.93, *SD* = 9.68, 95% CI (−3.35, 1.48)]. In order to verify that the attenuating effect *per se* was significant, we performed additional one-sample *t*-tests to determine if the scores differed from zero. The tests demonstrated that the attenuating score, *t*(63) = 2.36, *p* < 0.05, revealed a significant feedback effect, while the enhancing score did not differ from zero, *t* < 1.

### Smile Versus Frown

To compare the feedback effects between smiling and frowning, the four mean SFFE scores of each participant were collapsed into two mean scores, one for smiling and one for frowning. The data were analyzed with a one-way within subject ANOVA with the factor muscle (smile vs. frown). The result showed no effect of muscle, *F*(1,63) = 0.31, *MS*_e_ = 153.58, *ns*, *η*_*p*_^*2*^ = 0.01, and as seen in the middle panel of Fig. 3 there was no major difference in SFFE scores between when participants smiled [*M* = 1.07, *SD* = 10.11, 95% CI (−1.46, 3.59)] and frowned [*M* = 2.29, *SD* = 9.47, 95% CI (−0.08, 4.65)].

### Positive Versus Negative

To compare the feedback effects during positive emotions with the effects during negative emotions, the four mean SFFE scores of each participant were collapsed into a final set of two mean scores, one for positive emotions and one for negative emotions. To analyze the data, a one-way within subject ANOVA with the factor emotion (positive vs. negative) was performed. As can be seen in the right panel of Fig. 3 there was a non-significant trend towards slightly larger scores to positive [*M* = 2.77, *SD* = 6.60, 95% CI (1.12, 4.42)] than negative emotions [*M* = 0.59, *SD* = 6.43, 95% CI (−1.02, 2.19)], *F*(1,63) = 3.27, *MS*_e_ = 46.65, *p* < 0.10, *η*_*p*_^*2*^ = 0.05.

## Discussion

Our findings suggest that facial feedback primarily affects present emotions when the facial action is incongruent with the emotional valence. In fact, there were no indications of feedback effects at all when the facial action and the emotion were congruent. This absence of a feedback effect in enhancing modulation is unexpected considering that several previous studies have demonstrated enhancing effects (e.g., Soussignan 2002; Strack et al. 1988). One explanation for the diverging results could be that the present Experiment 2 was specifically designed to evaluate possible differences between enhancing and attenuating modulation. That is, in contrast to previous studies, the present study did not only let participants both smile and frown to positive and to negative stimuli, but instead, data were transformed and analyzed in a way making it possible to combine enhancing smiles and frowns, and combining attenuating smiles and frowns, in order to evaluate the overall enhancing and attenuating effects *per se*. It seems that this approach was effective in revealing attenuating effects. Still, since the attenuating effect in the present study was unexpected, it would be valuable with more research on the subject before drawing definitive conclusions. In light of this we realized that the data in the present Experiment 1 in fact could be transformed and reanalyzed in a way that would allow us to further test the main questions of Experiment 2. This reanalysis is presented in the Reanalysis of Experiment 1 section below.

Regarding the two other questions of the present study, no significant differences were found. That is, the acts of smiling and frowning appeared to produce feedback effects to a similar degree. Moreover, there was no significant difference in feedback effects during positive and negative emotions, which is in line with earlier results (Dimberg and Söderkvist 2011).

Finally, five participants in Experiment 2 realized the true purpose of the study, as compared to Experiment 1 in which no participants realized the true purpose. However, in the interviews after the experiment this was explained by the fact that these participants had heard about and were familiar with the facial feedback theory.

## Reanalysis of Experiment 1

If the significant difference in feedback effects between enhancing and attenuating modulation in Experiment 2 represents a genuine attenuating effect it should be possible to replicate this effect in other experiments where the SFFE scores for smiling and frowning can be calculated for enhancing and attenuating modulation. As it happens, and mentioned above, Experiment 1 in the present study can be considered such an experiment, which presents us with an immediate opportunity to test if the unexpected attenuating effect in Experiment 2 can be replicated.

That is, in the results of Experiment 1 it was demonstrated that there were no effects at all of facial feedback after 5 min and after 1 day. Consequently, these later conditions, and especially the condition after 1 day, could be assumed to reflect a more neutral muscle condition where participants were not influenced by voluntary performed facial actions. As detailed in Experiment 2, a neutral muscle condition makes it possible to discern the specific feedback effects of smiling and frowning, which in turn enable us to address the present questions.

Following the same procedure for transformations as in Experiment 2, and using the ratings after 1 day as the neutral muscle condition, new dependent variables were created in the form of SFFE scores for smiling and frowning. As a result of the transformations, four mean SFFE scores for each participant were created, that is, one score for smiling and one score for frowning during positive emotions, and one for smiling and one for frowning during negative emotions. Similar to Experiment 2, the three questions were evaluated by help of three different factors, one for modulation (enhancing vs. attenuating), one for muscle (smiling vs. frowning), and one for emotion (positive vs. negative), and the factors were analyzed in three separate one-factorial ANOVAs, with the data reconstructed for each analysis.

To compare the feedback effects between enhancing and attenuating modulation, the four mean SFFE scores of each participant were, similarly as in Experiment 2, collapsed into two mean scores, one for enhancing and one for attenuating modulation. The result demonstrated a significant effect of modulation, *F*(1,31) = 10.92, *MS*_e_ = 255.49, *p* < 0.05, *η*_*p*_^*2*^ = 0.26, with higher SFFE scores in attenuating [*M* = 10.31, *SD* = 16.40, 95% CI (4.40, 16.23)] as compared to enhancing modulation [*M* = −2.89, *SD* = 13.13, 95% CI (−7.63, 1.84)]. In order to verify that the attenuating effect *per se* was significant, we performed additional one-sample *t*-tests to determine if the scores differed from zero. The tests demonstrated that the attenuating score, *t*(31) = 3.65, *p* < 0.05, reflected a significant feedback effect, while the enhancing score did not differ from zero, *t* < 1.05.

Regarding the two other questions, there was no significant difference in SFFE scores between when the participants smiled [*M* = 6.60, *SD* = 11.92, 95% CI (2.30, 10.90)] and frowned [*M* = 0.82, *SD* = 13.20, 95% CI (−3.94, 5.58)], *F*(1,31) = 4.09, *MS*_e_ = 130.53, *p* < 0.10, *η*_*p*_^*2*^ = 0.12, and there was no significant difference during positive emotions [*M* = 4.75, *SD* = 12.00, 95% CI (0.42, 9.07)] or negative emotions [*M* = 2.67, *SD* = 14.44, 95% CI (−2.54, 7.88)], *F*(1,31) = 0.42, *MS*_e_ = 166.57, *ns*, *η*_*p*_^*2*^ = 0.01.

The results of this reanalysis demonstrate that facial feedback almost exclusively attenuated emotions, with quite a large effect size. This replicates the results of Experiment 2 and further confirms that facial feedback primarily affects ongoing emotions when the facial action is incongruent to the emotion. Just as in Experiment 2 there were no indications of any feedback effects when the facial action was congruent to the emotion.

## General Discussion

The purpose of the present study was to further detail the description of how facial feedback effects occur. In two experiments, four different questions were addressed. In Experiment 1, it was demonstrated that the effect of facial feedback primarily occurs at the same time when the facial action is performed. In Experiment 2 it was demonstrated that facial feedback primarily attenuates ongoing emotions, and the same pattern was evident in the Reanalysis of Experiment 1. Notably, there seems to be no systematic difference in feedback effects between smiles and frowns, or between positive and negative emotions.

Regarding the finding that facial feedback primarily attenuates present emotions there are a number of studies in the literature on facial reactions to emotional stimuli (e.g., Dimberg et al. 2000, 2002) that provide important insights. First, when people are exposed to emotional facial stimuli they spontaneously and unconsciously react with their corresponding facial muscles. These reactions are automatic, very quick, and not always visible to the eye, but can be detected with facial electromyographic equipment. Second, these automatic reactions affect voluntary facial actions by facilitating congruent actions. For example, people that are exposed to a positive stimulus react quickly and automatically with their smile muscles even before they are consciously aware of the valence of the stimulus. Then, if they are instructed to voluntarily react with a smile and/or a frown to the positive stimulus, the voluntary smile will be formed faster and be more pronounced than the voluntary frown that requires more time and is less pronounced. The same is true for a negative stimulus, which facilitates a faster and stronger voluntary frown, as compared to a voluntary smile. Therefore, since these automatic reactions are present even when people are instructed to not move their facial muscles, one could question if it is possible at all to expose participants to a truly neutral muscle condition in an experiment that uses emotional facial stimuli, such as in the present study. That is, when we compared the difference in feedback effects between voluntary facial actions and neutral muscle conditions, automatic facial reactions similar to those described above were probably initially present in all conditions. It is therefore interesting that even though a congruent facial action is faster and stronger, as compared to an incongruent action (Dimberg et al. 2002), it was the latter actions that primarily modulated ongoing emotional experiences.

In the facial feedback literature there are no obvious explanations for the attenuating effect found in the present study. One potential explanation could perhaps be found in the above mentioned field of automatic facial reactions to emotional stimuli (e.g., Dimberg et al. 2000, 2002), where recent studies (e.g., Wood et al. 2016a, b) suggest that incongruent facial actions can disrupt the automatic mimicry of facial stimuli and that this lack of mimicry might lower the general impact of the stimulus. If this is the case it could offer an explanation for the attenuating effect of incongruent facial actions, but it would not explain the absence of an enhancing effect from congruent actions.

Another potential explanation, based on the idea that emotions are governed by different affect programs (Tomkins 1962), could be that one function of emotional facial actions is to *initiate* corresponding affect programs. That is, if no emotions were present a specific facial action would initiate a corresponding affect program and thus initiate an emotional experience. With an emotion already present, however, a congruent facial action would probably have no or little effect since the corresponding affect program already would have been initiated by, for example, an emotional stimulus. An incongruent facial action, on the other hand, would initiate a conflicting affect program, which might reduce the activation of the originally initiated program. Admittedly, while this explanation would account for the present results it remains hard to test empirically.

Finally, there is also the possibility that the present attenuation effect is the result of a ceiling effect in the intensity of the induced emotions. In such a case, the presented stimuli evoked the emotions to a maximum level and simply left no room for further enhancement, but plenty of room for an incongruent action to cause attenuation. This is unlikely though since the present stimuli, pictures of facial expressions, are not expected to induce strong emotions (e.g., Wangelin et al. 2012). A suggestion for future research could be to investigate if there is any difference in general feedback effects, and between enhancing and attenuating modulation, when stimuli of different intensity levels are used. It would be interesting to see if the feedback effects become stronger with stimuli that induce very strong emotions, or if the effects even become weaker (e.g., Baumeister et al. 2016). Moreover, in order to eliminate the possibility of a ceiling effect it could also be interesting to use stimuli that is expected to induce weaker emotions, for example pictures of mildly happy and mildly angry persons, which would allow congruent actions to enhance the evoked emotions in case of a ceiling effect.

The effort by Wagenmakers et al. (2016) to replicate the well-known feedback study by Strack et al. (1988) is the largest and most ambitious replication study of facial feedback to this day. As discussed earlier, they could not replicate the original results which questions the original study, but it should also be noted that some of the methodological choices made in the replication study have been questioned by Strack (2016). It is also important to note, as Wagenmakers et al. (2016) do as well, that the failed replication does not mean a general refutation of the facial feedback hypothesis. Indeed, as the results of the present study also suggest, facial feedback effects have been demonstrated in numerous studies that cover a wide range of different methods. For this reason there is a need for more replication efforts and a continued systematic evaluation of the facial feedback field, and the scope and ambition of the effort by Wagenmakers et al. (2016) has certainly set the standard for future such studies.

Finally, the present results may have potential implications for the clinical studies that investigate the effect of Botox in the treatment of depression (e.g., Finzi and Rosenthal 2014; Magid et al. 2014; Wollmer et al. 2012, 2014). If, as we found in the present study, facial feedback primarily attenuates present emotions, and further, if constant frowning contributes to the depressed state, then this frowning may act to specifically attenuate positive emotions. The paralysis of frown muscles with Botox could then block this attenuation, which may result in a general recovery of positive emotions, and consequently this effect could be one basis for the documented improvements of the patients. Future research could study how Botox affects the day-to-day emotional life of depressed patients in order to find out in which situations the improvements are experienced most frequently.

The second question of Experiment 2 was if smiling or frowning produces a stronger feedback effect than the other. The results indicated that there were no significant differences in effect between them, which suggest that a smile and a frown produce feedback effects that are roughly equal in strength. That is, in relation to the specific emotional stimuli used in these experiments, they produced equally strong feedback. One interesting question is if the smile and the frown actions might differ in how easy they are to activate and/or differ in the strength of the activation itself. This question has earlier been addressed in studies that also used *the voluntary facial action technique* (for a discussion see Dimberg and Söderkvist 2011). In that study it was noted that the strength of the muscle activity for smiles and frowns did not differ and further that the activity levels over repeated trials were not differently affected by for example fatigue. This suggests that the two different facial actions should be comparable to each other. Moreover, the stimulus material consisted of clearly positive and clearly negative pictures, and it is possible that the relative strength between smiling and frowning could be different with another type of stimuli. Future research could therefore use more neutral or ambiguous stimuli to further investigate this question, and if completely neutral stimuli were used it would allow for a comparison of the initiating ability of facial feedback between smiling and frowning.

The third question of Experiment 2 was if positive or negative emotions are easier to modulate with feedback. This question has been addressed in both Dimberg and Söderkvist (2011) and in the present study, but none of these studies have found any significant differences between positive and negative emotions. There simply seems to be no difference between them in this regard.

In the present study it was assumed that the participants followed the instructions correctly. They were not monitored during the experiments, but we have no indications that they found the instructions difficult to follow. The control questions afterward aimed to find out if they had realized the true purpose, but also aimed to verify that they had followed the instructions. The few participants that did not follow the instructions correctly were easily identified. Note also that if a participant should not follow the muscle instructions correctly it would in fact work against the facial feedback hypothesis and consequently reduce the general level of facial feedback effect in the present experiments.

As mentioned above, the purpose of the present study was to further detail the description of how facial feedback occurs. The facial feedback hypothesis, as discussed by Adelmann and Zajonc (1989), defines facial feedback as facial muscle actions that have the ability to modulate present emotions or, in the absence of emotions, to initiate emotions. Collectively then, we would like to add that the effect of facial feedback mainly occurs at the time when the facial action is performed, and that facial feedback primarily attenuates ongoing emotional experiences.
